# The Association Between HLA Class II Alleles and the Occurrence of Factor VIII Inhibitor in Thai Patients with Hemophilia A

**DOI:** 10.5505/tjh.2012.29795

**Published:** 2012-03-05

**Authors:** Oytip Nathalang, Pramote Sriwanitchrak, Werasak Sasanakul, Ampaiwan Chuansumrit

**Affiliations:** 1 Thammasat University, Faculty of Allied Health Sciences, Department of Medical Technology, Pathumtani, Thailand; 2 Mahidol University, Faculty of Medicine, Ramathibodi Hospital, Department of Pediatrics, Bangkok, Thailand

**Keywords:** HLA class II alleles, FVIII inhibitor, Hemophilia A, Thais

## Abstract

**Objective:** This study aimed to investigate the association between HLA class II alleles and the occurrence of FVIIIinhibitor in Thai hemophilia A patients.

**Material and Methods:** The distribution of HLA-DRB1 alleles and DQB1 alleles in 57 Thai hemophilia A patientsand 36 blood donors as controls was determined using the PCR sequence-specific primer (PCR-SSP) method, and theassociation between the occurrence of factor VIII (FVIII) inhibitor and the presence of certain HLA class II alleles wasinvestigated.

**Results:** The frequency of HLA-DRB1*15 was higher in the hemophilia A patients with and without FVIII inhibitor,whereas that of DRB1*14, DRB1*07, and DQB1*02 was lower in the hemophilia A patients with FVIII inhibitor, ascompared to controls. Interestingly, only the frequency of DRB1*15 was significantly higher in the patients with inhibitorthan in the controls (P = 0.021). Moreover, the frequency of DRB1*15 in the patients with inhibitor was higher than inthose without inhibitor (P = 0.198).

**Conclusion:** The study’s findings show that the DRB1*15 allele might have contributed to the occurrence of inhibitorin the Thai hemophilia A patients; however, additional research using larger samples and high-resolution DRB1 typingis warranted.

## Abstract (tr)

**Amaç:** Bu çalışmada HLA sınıf II allelleri hemofili A ile Tayland’lı hastalarda FVIII inhibitörü oluşumu ile ilişkiyi araştırmaktır.

**Gereç ve Yöntemler:** Hemofili 57 Tayland’lı hastalarda HLA-DRB1 allelleri ve DQB1 allel dağılımı bir PCR-sıra özel astar (PCR-SSP) yöntemi ve faktör VIII (FVIII) inhibitör oluşumu arasındaki ilişki kullanılarak belirlendi ve bazı HLA klas II allelleri varlığı araştırılmıştır.

**Bulgular:** HLA-DRB1 sıklığı * 15 ve FVIII inhibitörü olmayan iki hemofili hastalarında artmış iken DRB1 olanlar * 14, DRB1 * 07 ve DQB1 * 02 FVIII inhibitörleri ile hemofili hastalarında azalmıştı. İlginçtir, sadece DRB1 * 15 önemli kontrol (P = 0.021) ile karşılaştırıldığında inhibitörleri olan hastalarda artmıştır. Ayrıca, inhibitörü olan hastalarda DRB1 * 15 frekans olmayan inhibitörü (P = 0.198) olan hastalarda daha yüksek olma eğiliminde.

**Sonuç:** Bulgularımız DRB1 * 15 allel Tay hemofili A hastalarında inhibitör oluşumuna katkıda bulunabileceğini göstermiştir. Ancak, daha büyük bir örneklem büyüklüğü ve yüksek çözünürlüklü DRB1 yazarak garantilidir.

## INTRODUCTION

One of the most serious complications in patients with hemophilia A is the occurrence of an inhibitor frequently,IgG4 antibodies directed against epitopes in factor VIII(FVIII). This may be induced by replacement of the missing factor via cryoprecipitate or FVIII concentrate administration.The antibody attached to FVIII will neutralize orinhibit its ability to stop bleeding. FVIII inhibitor is usuallydetected in one of two ways. First, the inhibitor maybe discovered in asymptomatic patients via routine screening performed during a comprehensive clinical examination.Second, an inhibitor may be detected when bleedingis suddenly and unexpectedly unresponsive to treatment with FVIII. Generally, the incidence of FVIII inhibitor in hemophilia A patients with severe disease (FVIII:C <1%)and moderate disease (FVIII:C >1% to 5%) is estimated to be 20% and 33%, respectively [1,2,3,4,5,6]; however, differences in the incidence between ethnic groups might be due togenetic differences. Moreover, the lack of recognition isone of the causes of low incidence of inhibitor in economicallyless-developed countries.

It has been reported that molecular defects in thefactor VIII gene and the major his to compatibility complex molecules, especially HLA class II alleles, are associated with antibody formation. An increased occurrence of FVIII inhibitor was reported in cases of severe congenital hemophilia A with HLA-DRB1*15:01, DQA1*01:02,and DQB1*06:02 alleles [8,9]. Conversely, another study reported that HLA class I alleles were not associated withthe occurrence of FVIII inhibitor in patients with acquired hemophilia A, whereas DRB1*16 and DQB1*0502 were associated with a high risk of such an occurrence in hemophilia A patients with FVIII inhibitor [10]; however,the association between these alleles and FVIII inhibitor in Thai patients with hemophilia A remains unknown. Assuch, the present study aimed to investigate the association between HLA class II alleles and the occurrence of FVIII inhibitor in a group of Thai patients with hemophilia A.

## MATERIALS AND METHODS

The study included 57 hemophilia A patients from Mahidol University, Faculty of Medicine RamathibodiHospital, Division of Hematology, Department of Pediatrics,Bangkok, Thailand, and a control group consisting of36 unrelated male blood donors from the National BloodCenter of the Thai Red Cross Society. The study protocolwas approved by the Mahidol University, Faculty of Medicine Ethics Committee and the Committee on Human Rights Related to Research Involving Humans. Informedconsent was obtained from each participant and/or his parents.

The patients were regularly monitored for factor VIIIinhibitor every 6-12 months or when clinically indicatedin cases of unresponsiveness to replacement therapy. The inhibitor titer against human factor VIII clotting activitywas determined via the Bethesda method [11]. A Bethesdaunit (BU) level >0.6 was considered indicative of the presence of inhibitor. Moreover, genetic defect associated with hemophilia A was carried out. Inversion of intron 22 wasinitially determined via inverse polymerase chain reaction(PCR) [12,13]. In patients without inversion of in tron 22conformation-sensitive gel electrophoresis was used tofurther investigate the genetic defect [14], followed bysequencing.

Genomic DNA was extracted from peripheral bloodcells using the salting out technique [15]. The secondexon of the DRB1 and DQB1 genes was amplified using the PCR-SSP method. Each DNA sample (100 ng μL–1)was tested using a Micro SSP Generic HLA Class II TypingKit (One Lambda Inc., Canoga Park CA, USA). Briefly, for HLA class II low-resolution typing each DNA sample (100ng) was amplified with 31 different primer sets optimized and dispensed into each well of a 96-well thin-walled PCR plate. The SSP-DNA reaction set was placed in a G-STORMGS1 thermal cycler (Gene Technologies Ltd., Essex, UK).The cycle parameters of the PCR program were set accordingto the manufacturer’s instructions. The reaction patternwas photographed and HLA alleles were assessed via analysis of the gel banding pattern using a reaction pattern typing grid.

The association of the HLA class II alleles and thedevelopment of an inhibitor in Thai patients with hemophilia A was calculated using the odds ratio (OR) and 95%confidence interval (CI). The frequency of alleles in thepatients and controls was compared using chi-square contingency table analysis with Yates’ correction, as well as standard P values and Fisher’s exact test. A P value <0.05was accepted as statistically significant.

## RESULTS

The study included 57 male Thai hemophilia A patientswith a mean age of 14.4 ± 8.9 years. The patients weredivided into 2 groups: 26 patients without inhibitors and31 patients with a high inhibitor titer ≥5 BU (n = 22), lowinhibitor titer <5 BU (n = 3), and transient low inhibitortiter for <6 months (n = 6). The mean high inhibitor titerwas 540.9 BU (range: 5.3-3920 BU) and the mean lowtiter was 3.3 BU (range: 2.9-4.2 BU), whereas the meantransient low titer was 2.0 BU) (range: 1.0-3.3 BU).

A molecular defect related to the factor VIII gene wasobserved in 35 of the 57 patients (61.4%) of which 15 were in the non-inhibitor group and 20 were in the inhibitorgroup. In all, 6 patients in the non-inhibitor group and12 patients in the inhibitor group had inversion of intron22: however, the difference in the number of patients withinversion between the patients with and without inhibitorwas not statistically significant (P = 0.32). The specificmutations were investigated in the 17 patients withoutinversion of intron 22; 10 patients had point mutationsand mutations could not be identified in the other7 patients. Interestingly, point mutations inducing stopcodon (n = 2), amino acid alteration (n = 2), and frameshiftmutation (n = 1) were observed in patients withoutinhibitor, and point mutations inducing stop codon (n =5) were noted in patients with inhibitor. The occurrenceof stop codon in patients with inhibitor was higher thanthose without inhibitor (P = 0.05).

The distribution of HLA-DRB1 and DQB1 alleles,according to PCR-SSP low-resolution typing, in the patientswith and without inhibitor, and in the controls is shown inTables 1 and 2. Overall, 13 DRB1 alleles were noted in thehemophilia A patients, of which DRB1*15 and DRB1*12were the most frequent; additionally, 7 DQB1 alleles wereidentified. The most common DQB1 alleles in the patients and controls were DQB1*05 and DQB1*06, respectively.DQB1*03 was sub-typed as DQB1*03:01/03:04,DQB1*03:02/03:05/03:07, and DQB1*03:03:02/03:06via PCR-SSP low-resolution typing.

The frequency of DRB1*15 was higher in the patients(both with and without inhibitor) than in the controls,however, statistical significance was found betweenpatients with inhibitor and the controls (30.6% vs.13.9%; P = 0.021; OR = 2.74; 95% CI = 1.16-6.47). Thefrequency of DRB1*15 in the patients with FVIII inhibitor(30.6%) was higher than that in the patients withoutinhibitor (19.2%), but the difference was not statisticallysignificant (P = 0.198). On the other hand, the frequencyof DRB1*14, DRB1*07, and DQB1*02 was lower in thepatients with inhibitor than in those without inhibitor (P> 0.05).

## DISCUSSION

Both genetic and non-genetic risk factors have beenimplicated in the development of factor VIII inhibitor[16,17]. The molecular defects in the FVIII gene that causea defect in translation and protein production is a primarycause of inhibitor formation. Polymorphisms associatedwith HLA class II molecules, interleukin-10 (IL-10), andtumor necrosis factor-a (TNF-a) also influence to theFVIII inhibitor development [18]. Immunological mechanismsin the cellular processing of peptide antigens areinvolved in the development of inhibitor in patients withhemophilia A. Moreover, the major histocompatibilitycomplex phenotype is also involved in inhibitor formation[19-21].

Although a molecular defect associated with factor VIIIwas observed in 35 of the 57 patients in the present study,inversion of intron 22 was observed in more of the patientswith inhibitor than in those without inhibitor, as previouslyreported [22,23]; however, the frequency of point mutationscausing stop codon in patients with inhibitor wassignificantly higher than in those without inhibitor, whichwas also previously reported [24]. Moreover, the associationbetween HLA class II alleles and the FVIII inhibitorsin Thai hemophilia patients was further investigated inthis study, as recent studies have indicated that inhibitorformation depends upon an adequate T-cell responseby major histocompatibility complex class II moleculesto FVIII resulting from the presentation of FVIII proteinantigen to T-cell receptors [18-21]. It was reported that inmild hemophilia A patients with inhibitor the frequencyof DRB1*01 and DQB1*05 was slightly higher than thecontrols (but not significantly) [25]. A comparison of thefrequency data for DRB1*15/16 in hemophilia A patientswith FVIII inhibitor reported in other studies showed thatDRB1*15 and DRB1*16 were high-risk alleles for inhibitorformation in patients with congenital hemophilia Aand acquired hemophilia A, respectively [8-10].

The DRB1*15 allele is known to exhibit the specificsurface loop peptide comprising amino acids 1706-1721of the FVIII light chain, and is considered to be involvedin FVIII inhibitor formation in patients with congenitalhemophilia A that lack endogenous FVIII protein synthesis[8,26]. Because the ability to recognize and processFVIII peptides is determined by the number of HLAclass II molecules in each individual. It was reported that there are as many as 13 potential recognition sequencesfor HLA-DRB1*1501 in FVIII, whereas there are only 2recognition sequences for HLA-DRB1*1101 [27]. Eventhough HLA-DRB1*15 (17.5%), DRB1*12 (16.9%), andDRB1*09 (11.5%) were the most common in Thai blooddonors [28], the frequency of the DRB1*15 allele amonghemophilia A patients with inhibitors in the present studywas significantly higher than in the controls.

Limitations of the present study included the smallnumber of patients enrolled, incomplete detection ofmolecular defects of the factor VIII gene, and the lack ofexploration of the polymorphisms associated with IL-10and TNF-a. In conclusion, the DRB1*15 allele may havecontributed to inhibitor formation in Thai patients withhemophilia A. Additional comprehensive research withlarger patient populations is warranted.**Conflict of Interest Statement**The authors of this paper have no conflicts of interest,including specific financial interests, relationships, and/or affiliations relevant to the subject matter or materialsincluded.

## ACKNOWLEDGEMENTS

This study was supported by the Thailand ResearchFund: Senior Research Scholar 2006 (AC).

## Figures and Tables

**Table 1:**
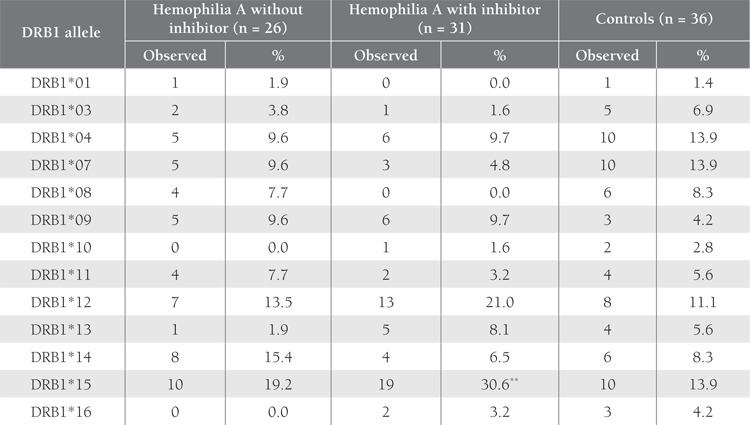
Distribution of HLA-DRB1 Alleles in the Thai Hemophilia A Patients and Controls

**Table 2:**
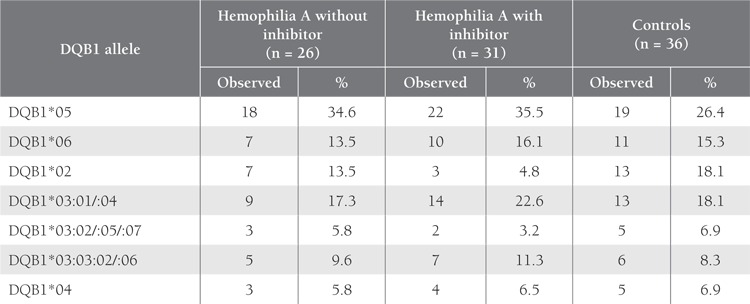
Distribution of HLA-DQB1 Alleles in the Thai Hemophilia A Patients and Controls
